# The German version of the Mini Suffering State Examination (MSSE) for people with advanced dementia living in nursing homes

**DOI:** 10.1186/s12877-022-03268-0

**Published:** 2022-07-18

**Authors:** Naomi Zumstein, Keiko Yamada, Stefanie Eicher, Nathan Theill, Heike Geschwindner, Henrike Wolf, Florian Riese

**Affiliations:** 1 University Research Priority Program “Dynamics of Healthy Aging”, University of Zurich, Andreasstrasse 15, 8050 Zurich, Switzerland; 2 Department of Anthropology, McGill University, 855 Sherbrooke Street West, Montreal, QC H3A 2T7 Canada; 3 Department of Psychology, McGill University, 2001 McGill College Avenue, Montreal, QC H3A 1G1 Canada; 4 Department of Anesthesiology and Pain Medicine, Faculty of Medicine, Juntendo University, 2-1-1 Hongo, Bunkyo-ku, Tokyo, 113-8421 Japan; 5 Center for Gerontology, University of Zurich, Pestalozzistrasse 24, 8032 Zurich, Switzerland; 6 Division of Geriatric Psychiatry, University Hospital of Psychiatry, Lenggstr. 31, 8032 Zurich, Switzerland; 7 City of Zurich Nursing Homes, Eggbühlstrasse 23, 8050 Zurich, Switzerland; 8 Psychiatrische Dienste Graubünden, Ambulatory Psychiatric Services, Piazza Paracelsus 2, 7500 St. Moritz, Switzerland

**Keywords:** Advanced dementia, End-of-life care, Mini Suffering State Examination (MSSE), Palliative care, Suffering, Validation

## Abstract

**Background:**

The Mini Suffering State Examination (MSSE) has been explicitly recommended to assess suffering in dementia patients. This study aimed to develop a German version of the MSSE and assess its psychometric properties involving people with advanced dementia (PAD) in a nursing home setting.

**Methods:**

The MSSE was translated into German, and 95 primary nurses administered it cross-sectionally to 124 PAD in Zurich, Switzerland. The psychometric properties of the German MSSE version were calculated for this population.

**Results:**

The mean age of the PAD was 83.3 years (SD = 9.1, range = 55–102 years), and 98 of them (79.0%) were women. The Kuder-Richardson Formula 20 coefficient for the entire scale (0.58), the eight items relating to objective health conditions (0.39), and the professional and family estimation of the patient’s suffering (0.64) indicated low internal consistency. A confirmatory factor analysis indicated an unsatisfactory fit to a one-factor structure, with a comparative fit index and root mean square error of approximation of 0.71 and 0.08, respectively, and a Tucker–Lewis index of 0.64. The MSSE total score was significantly but moderately correlated with the total scores of the Symptom Management–End-of-Life with Dementia (SM-EOLD) scale (Pearson’s correlation coefficient (r)** = -**0.44; *p* < 0.05), the physical suffering scores (*r* = 0.41; *p* < 0.05), and the psychological suffering scores (*r* = 0.55; *p* < 0.05).

**Conclusions:**

The German version of the MSSE questionnaire did not perform well in the nursing home setting involving PAD. The instrument had low internal consistency, doubtful validity, and could not discriminate between suffering and other distressing symptoms. We do not recommend its use in this population.

## Background

People with advanced dementia (PAD) experience an increase in burdensome symptoms, such as pain, agitation, shortness of breath [1–3], and high levels of physical, psychological, and existential or spiritual suffering toward the end of life [4, 5]. Pain and other distressing symptoms are not synonymous with suffering; they are phenomenologically distinct [6]. For a holistic approach to caring for PAD, it is essential to systematically discriminate between suffering and other distressing symptoms [6, 7].

Suffering is a complex, difficult-to-assess phenomenon. Consequently, self-reporting is the most reliable method for determining its intensity and magnitude [8]. As cognitive and verbal communication abilities in PAD tend to be severely impaired [9, 10], suffering must be assessed by proxy respondents, a fair substitute in nursing home settings [11].

The Mini Suffering State Examination (MSSE) scale was specifically developed to assess suffering in dementia patients at the end-of-life stage [12]. It has been explicitly recommended to measure the quality of dying in populations with dementia and in mixed long-term care populations in nursing or residential care home settings [13]. The instrument’s developers suggested that it has performed well with people with “end-stage dementia” in Israel [12, 14]. Another study maintained that there is a valid Dutch MSSE version available and that it performed well when used to measure the quality of dying among long-term care residents with dementia at the end-of-life stage [15]. However, the study cited by the authors as a reference for a valid Dutch MSSE version [16] only translated the MSSE into Dutch but did not assess the reliability and validity of the scores produced by this instrument.

A recent review of various suffering measurement instruments revealed that the study’s methodological quality in assessing the validity of the original English version of the MSSE questionnaire was doubtful and that the quality of the evidence was moderate [17]. The MSSE questionnaire used in the development cohort in Israel [12] produced inadequate internal consistency and doubtful reliability and criterion validity [17]. In addition, no assessment of unidimensionality was carried out by using confirmatory factor analysis (CFA) as recommended by Streiner [18]. Thus, it is unclear if the MSSE measures the construct of suffering as intended.

A study assessing the modified Spanish MSSE version suggested that content validity was acceptable and internal consistency moderate when used to measure suffering among patients with advanced cancer [19]. However, the MSSE was initially developed to assess suffering among patients with end-stage dementia, and the results of the Spanish study are not directly transferable to this population. Also, the questionnaire was administered one to six months after the patient’s death which might have led to recall bias. In addition, the authors did not assess structural validity by conducting a confirmatory factor analysis (CFA). Hence, it is difficult to judge if the Spanish version measures the construct of suffering as supposed.

Another recent systematic review and narrative synthesis [20] criticized the selection criteria used for the development cohort. The MSSE developers combined the Functional Assessment Screening (FAS) Tool stage 7c [21] and the Mini-Mental State Examination (MMSE) score of 0/30 [22]. The authors of the review argue that the MMSE (which is not a dementia staging tool but a screening tool for possible cognitive impairment [23]) shows a floor effect when used in advanced dementia. Hence, the MMSE may not be appropriate for assessing cognition at the end of life in dementia, as it subsumes all presentations of severe dementia into a zero score. On the other hand, the FAS categorizes end of life in advanced dementia into five sub-stages (stages 7a to 7e) [24]. Yet, the FAS is not without its limitations. It assumes a sequential pattern of deterioration in people with dementia [25, 26] and, thus, does not account for the large heterogeneity in dementia trajectories [27].

Hence, different conceptual and methodological problems with the original MSSE version might have influenced the Dutch and Spanish versions. Nevertheless, the measurement properties of test scores are population- and situation-dependent [18]. A test may perform excellently in one context and poorly in another as the reliability and validity of the scores are influenced by the interaction between the test and the particular group completing it [28]. There is no extensively validated instrument in German to assess the level of suffering in PAD. This study aimed to translate and cross-culturally adapt the MSSE scale and assess the measurement properties of the German version in nursing homes.

## Methods

### Study population

The present study was part of the Zurich Life and Death with Advanced Dementia (ZULIDAD) study, the aim of which was to describe the situation of PAD dying in nursing homes [29]. It was a prospective, multi-perspective, observational study conducted in 11 nursing homes in the Zurich area of Switzerland: 10 municipal nursing homes and one privately managed nursing home specializing in dementia care (Sonnweid AG). Details of the recruitment process are available in the published study protocol [29]. The residents in the participating institutions were screened using the nursing homes’ Resident Assessment Instrument-Minimum Data Set (RAI-MDS) (Swiss version 2.0) databases [30]. The RAI tool was developed to provide a standardized and inter-disciplinary approach to care planning in long-term care settings. It consists of several modules, such as the MDS, which contains indicator elements, including disease diagnoses, health conditions, cognitive abilities, nutritional status, etc. Another element is the cognitive performance scale (CPS), which was developed from five MDS items (“comatose,” “short-term memory,” “cognitive skills for decision making,” “making self understood,” and “self performance”). The final CPS score ranges from 0–6, with higher scores indicating more severe cognitive impairment [23].

The inclusion criteria were a diagnosis of dementia (RAI-MDS items “Alzheimer’s disease” or “dementia other than Alzheimer’s disease”) and a CPS score of 5 or 6, which indicates severe impairment (“advanced dementia”) [23]. A CPS score of 5 is comparable to a Mini Mental State Examination (MMSE) score of 5 [31]. The exclusion criterion was cognitive impairment due to other conditions, such as a major stroke, tumor, or coma (RAI-MDS item “disease diagnoses”).

Of the 1,786 eligible nursing home residents, 410 (22.9%) met the inclusion criteria. Two of the 410 residents were not eligible due to other neurodegenerative diseases; 37 had no healthcare proxies to be contacted; and 15 died between the eligibility assessment and the start of the data collection. Altogether, 356 healthcare proxies (relatives and professionals) were contacted, with 126 (35.4%) consenting to participation.

### Questionnaires and data collection

The MSSE consists of eight items relating to health conditions and two items relating to professional and family estimations of the patient’s suffering. Each item has a binary score of present (1) or not (0) (yes/no format). The final score ranges from 0–10 (0–3 corresponding to low, 4–6 to intermediate, and 7–10 to high levels of suffering) [12]. Since no manual exists for the MSSE, no specific rater training could be performed with the nurses. All nurses underwent a 60–90 min one-on-one introduction with a researcher covering the study questionnaire. Furthermore, researchers were available throughout the study for all questions regarding the questionnaire. Although the Kuder-Richardson Formula 20 (KR-20) is a relevant index to evaluate internal consistency in dichotomously scored scales such as the MSSE, the instrument developers reported Cronbach’s alpha reliability coefficient in the original English version of the MSSE as 0.735 and 0.718, respectively, for groups assessed by two physicians [12].

In addition to the MSSE, symptom management was assessed with the Symptom Management–End-of-Life with Dementia (SM-EOLD) scale [32] and global estimations of physical and psychological suffering, with two separate single items to test for construct validity. The SM-EOLD consists of nine items, including those relating to pain, shortness of breath, and fear, and is recommended for assessing nursing home residents with dementia [33]. Proxy respondents indicate how frequently they have observed the symptoms in the last four weeks on a six-step scale (“never,” “once a month,” “two or three times a month,” “once a week,” “two or three times a week,” or “daily”), with possible scores ranging from 0 to 45 and higher scores indicating better symptom management. Global physical and psychological suffering were assessed using two separate questions referring to the previous seven days on an 11-step scale ranging from 0 (no suffering) to 10 (highest possible suffering): “How would you rate the extent of the PAD’s physical suffering?” “How would you rate the extent of the PAD’s psychological suffering?”.

The MSSE, the SM-EOLD, and the two separate questions assessing global physical and psychological suffering were all administered by primary nurses. The primary nursing care system emphasizes person-centered delivery and assigns specific nurses to specific patients [34]. Due to the close relationship that primary nurses develop with their patients, they can be considered reasonably accurate observers of suffering in PAD.

Of the 126 PAD included in the ZULIDAD study, data for two of them were missing because their primary nurses did not carry out the baseline measurements. Thus, 124 PAD were assessed by 95 primary nurses. Among them, 72 were responsible for one PAD each and 23 for multiple PAD (17 primary nurses for two PAD each and six primary nurses for three PAD each). Of the 124 PAD, SM-EOLD scores were missing for 10 (8.05%) of them. Thus, SM-EOLD scores were available for 114 PAD. The global estimations of physical and psychological suffering were available for all 124 PAD. (All data presented in this article are from baseline measurements collected between December 2013 and December 2014. Permission was obtained from the original author of the MSSE (Dr Aminoff) and of the SM-EOLD (Dr Volicer) to use and translate the instruments for this study.)

### German translation of the MSSE

The English version of the MSSE [12] was translated into German and cross-culturally adapted to the German context based on the International Society for Pharmacoeconomics and Outcomes Research (ISPOR) guidelines [35]. Two researchers performed independent forward translations and reconciled them into one forward translation, which was back-translated by a native English speaker with a professional background in dementia care. All three translators reviewed the back-translation and harmonized the German version with it. Three experts reviewed the harmonized German version—a general practitioner, a senior long-term care nurse, and a family member of a PAD—for comprehensibility, relevance, face validity, and intertranslation validity and finalized it.

### Psychometrical scale performance analysis

Scale development involves complex and systematic procedures grounded in theoretical and methodological rigor in relation to the measurement problem at hand. The theoretical model serves as a guide for conceptual formulations and the definition and operationalization of the phenomenon to be measured [36]. To assess whether the MSSE questionnaire matched the intended goals of the developers, we first analyzed the theoretical and conceptual framework of the MSSE questionnaire, how the latent construct of suffering was defined and operationalized, and whether the item generation was based on deduction, induction, or a combination of the two methods.

Clinically useful measures should exhibit minimal floor and ceiling effects, which are considered to be present when more than 15% of the persons assessed achieve the lowest or highest possible total score, respectively [37]. Consequently, patients with the lowest (0) or highest (10) possible total MSSE scores cannot be distinguished from each other concerning their level of suffering.

Structural validity was evaluated by confirmatory factor analysis (CFA) to assess whether the scores of the MSSE instrument would confirm the predefined unidimensionality of the construct of suffering [38]. We also conducted a Mokken scale analysis (MSA) [39] to assess the assumption of unidimensionality. The investigation of MSA models is suitable when the number of items in a questionnaire is low [40], as with the MSSE.

Construct validity—the degree to which the scores of an instrument are consistent with hypotheses based on the assumption that the instrument validly measures the intended construct—was assessed using convergent validity and divergent validity [41]. Convergent validity assesses how a scale correlates with related variables or other related measures, and divergent validity is an assessment of a scale’s lack of correlation with dissimilar variables or unrelated measures [18].

For convergent validity, we hypothesized that good correlations between the total MSSE score and global estimations of physical and psychological suffering would be found. For divergent validity, we hypothesized that a weak correlation between the total MSSE score and the SM-EOLD total scores would be found.

### Statistical analysis

All calculations were computed with STATA, version 16.1 for Mac [42]. Missing data were not imputed, and omitted items were excluded from the analysis. The measurement properties of the scores produced by the instruments were assessed using several indices [38, 43].

Descriptive analysis was used to calculate the mean scores (M) and standard deviations (SD) of the sociodemographic and clinical variables of PAD, the sociodemographic variables of the primary nurses, the MSSE total scores, the SM-EOLD total scores, and the two separate single items for the global estimations of physical and psychological suffering. Nominal data were reported as frequencies (numbers, percentages).

The internal consistency reliability of the dichotomously scored MSSE items was measured using the Kuder-Richardson Formula 20 (KR-20). A value of > 0.7 was expected [18, 44].

To assess structural validity, a CFA was used. Variation and covariation among the 10 items were evaluated using fit indices for a reflective one-factor structure model. We calculated the following fit indices: the root mean square error of approximation (RMSEA) with a 90% confidence interval (CI), the comparative fit index (CFI), and the Tucker–Lewis index (TLI). Although cutoff rules are still under discussion, it has been suggested that a CFI of > 0.95 and an RMSEA of < 0.06 indicate an acceptable fit for binary variables [45]. TLI values close to 0.95 are considered to demonstrate an acceptable fit. A factor loading of > 0.5 was expected for each item. Scalability was measured using Loevinger’s coefficient H. By convention, the strength of a scale is considered weak (0.3 ≤ H < 0.4), moderate (0.4 ≤ H < 0.5), or strong (0.5 ≤ H ≤ 1.0) [40].

Convergent and discriminant validity were determined by analyzing Pearson’s correlation [38]. The size of the correlational effects is considered small (0.1 < Pearson’s correlation coefficient (r) < 0.3), moderate (0.3 < *r* < 0.5), or high (*r* > 0.5) [46]. The level of significance (*p* value) of < 0.05 (two-tailed test) was considered statistically significant.

## Results

**Fig. 1:**
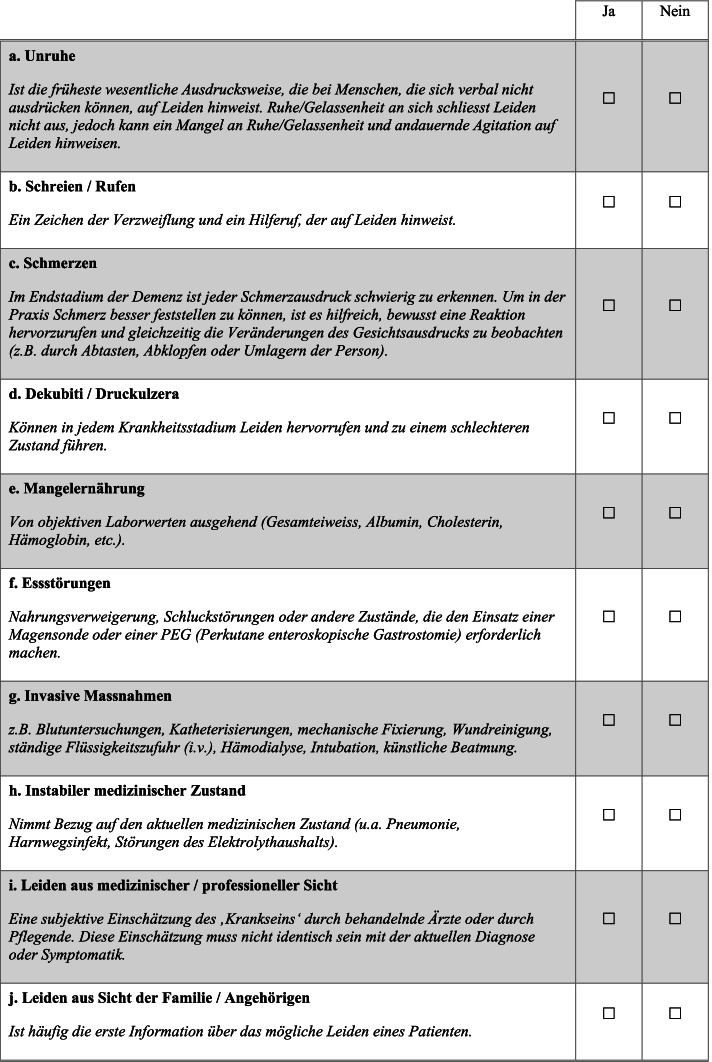
German version of the Mini Suffering State Examination (MSSE) questionnaire

No significant problems occurred during the translation of the MSSE. Compared with the original English version, no items were deleted from or added to the German version. The expert panel assessed the items’ intents and linguistic and cultural nuances. Minor changes were made to the wording for better comprehensibility, harmonizing the new translation with the source version and ensuring linguistic and cultural relevance. Thus, the linguistic validity of the German version was confirmed (see Fig. 1).

The developers of the original MSSE questionnaire asserted that the instrument’s conceptual model and theoretical framework were based on Cassel’s concept of suffering, which emphasizes its multidimensionality and threat to people’s integrity [47]. However, the instrument’s developers did not specify how Cassel’s definition of suffering was considered during the development of the original MSSE questionnaire, which theoretical model was used for item generation, why particular characteristics of suffering were chosen, and how they related to the target construct of suffering. Social, spiritual, and existential factors that are essential aspects of suffering as well as physical and psychological dimensions [6, 48] were omitted from the original MSSE questionnaire.

The mean age of the 124 PAD was 83.3 years (SD = 9.05, range = 55–102 years), and 98 of them were women (79.0%). Sixty-four PAD (51.6%) were diagnosed with Alzheimer’s disease, 56 (45.2%) with dementia other than Alzheimer’s disease, and four (3.2%) with both Alzheimer’s and dementia other than Alzheimer’s disease. All PAD had a CPS of 5, except two who had a CPS of 6, indicating that all PADs were severely impaired in their daily decision-making.

Of the 95 primary nurses involved in the evaluation process, 77 (81.1%) were women. The mean age was 45.1. Only three (3.2%) were certified in palliative care; 25 (26.3%) had external training in palliative care; 32 (33.7%) had internal training only; and 35 (36.8%) had no specific training in palliative care. The characteristics of the primary nurses, including their work experience in dementia care and the frequency of their contact with the PAD, are depicted in Table 1. The general characteristics of the German MSSE items are depicted in Table 2.

**Table 1:** Characteristics of primary nurses involved in the evaluation process (*N* = 95)

Age (years)	*M* = 45.1, *SD* = 11.8, Min. 19, Max. 63		
Sex	Women:	77	(81.1%)
	Men:	18	(18.9%)
Education in palliative care	Certified in palliative care	3	(3.2%)
	External training	25	(26.3%)
	Internal training only	32	(33.7%)
	No specific training	35	(36.8%)
Work experience in dementia care	> 15 years	34	(35.8%)
	10–15 years	16	(16.8%)
	5–10 years	17	(17.9%)
	1–5 years	24	(25.3%)
	< 1 year	4	(4.2%)

*M* Mean scores*, Min* Minimum*,* *Max* Maximum*, SD* Standard deviation

**Table 2:** Characteristics of the German MSSE items (*N* = 124)

Item	Frequencies “yes” (%)	Mean (SD)	Missing data rate (%)	Factor Loading	Loevinger’s Coefficient (H)
Not calm	72 (58.1)	0.58 (0.49)	–	0.19	0.57
Screams	31 (25.0)	0.25 (0.43)	0.8	0.20	0.57
Pain	50 (40.3)	0.40 (0.49)	–	0.44	0.43
Decubitus ulcers	10 (8.1)	0.08 (0.27)	–	0.31	0.43
Malnutrition	17 (13.7)	0.14 (0.35)	0.8	0.37	0.30
Eating disorders	24 (19.4)	0.19 (0.39)	–	0.24	0.38
Invasive action	5 (4.0)	0.04 (0.19)	–	0.09	0.28
Unstable medical condition	12 (9.7)	0.09 (0.29)	–	0.19	0.38
Suffering according to medical opinion	34 (27.4)	0.27 (0.44)	–	0.77	0.45
Suffering according to family opinion	34 (27.4)	0.28 (0.45)	3.0	0.59	0.36

*SD* Standard deviation

**Fig. 2:**
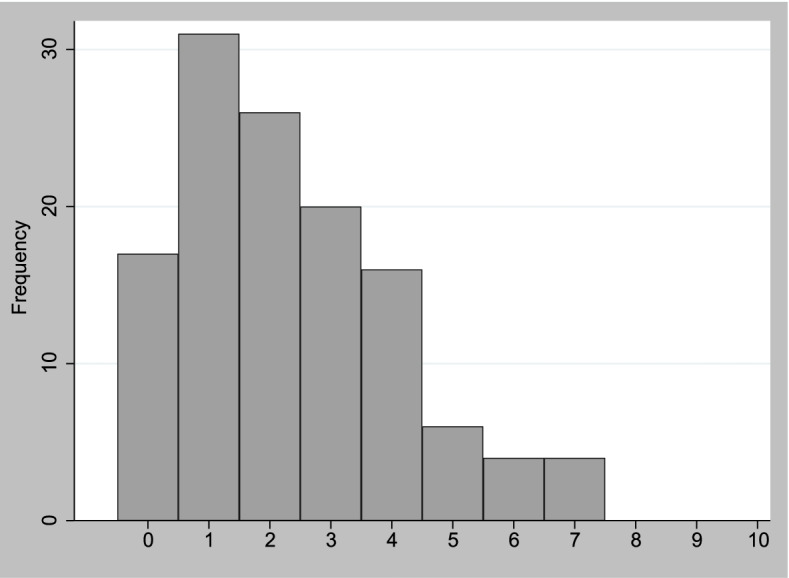
Distribution of total scores of the German MSSE. Legend: N = 124, Mean scores = 2.33, Standard deviation = 1.77; Interpretation of total scores. Aminoff et al. 2004 [12]: 0–3(low level of suffering), 4–6 (intermediate level of suffering), 7–10 (high level of suffering)

Of the 124 PAD, 94 (75.8%) had a low level of suffering; 26 (21.0%) had an intermediate level of suffering; and 4 (3.2%) had a high level of suffering. The mean total MSSE score for the 124 PAD was 2.33 (SD = 1.77). The distribution of the total MSSE scores showed the existence of floor effects as the PADs were clustered at the low level of suffering (see Fig. 2).

The mean total SM-EOLD score for the 114 PAD was 21.91 (SD = 9.16, range = 0–40), 2.29 (SD = 1.80, range = 0–10) for physical suffering, and 3.19 (SD = 2.35, range = 0–8) for psychological suffering of 124 PAD.

The KR-20 coefficient for the entire sample was 0.58 (0.56 for women and 0.67 for men), 0.39 for the eight items relating to health conditions, and 0.64 for the professional and family estimation of the patient’s suffering, indicating low internal consistency reliability.

**Fig. 3:**
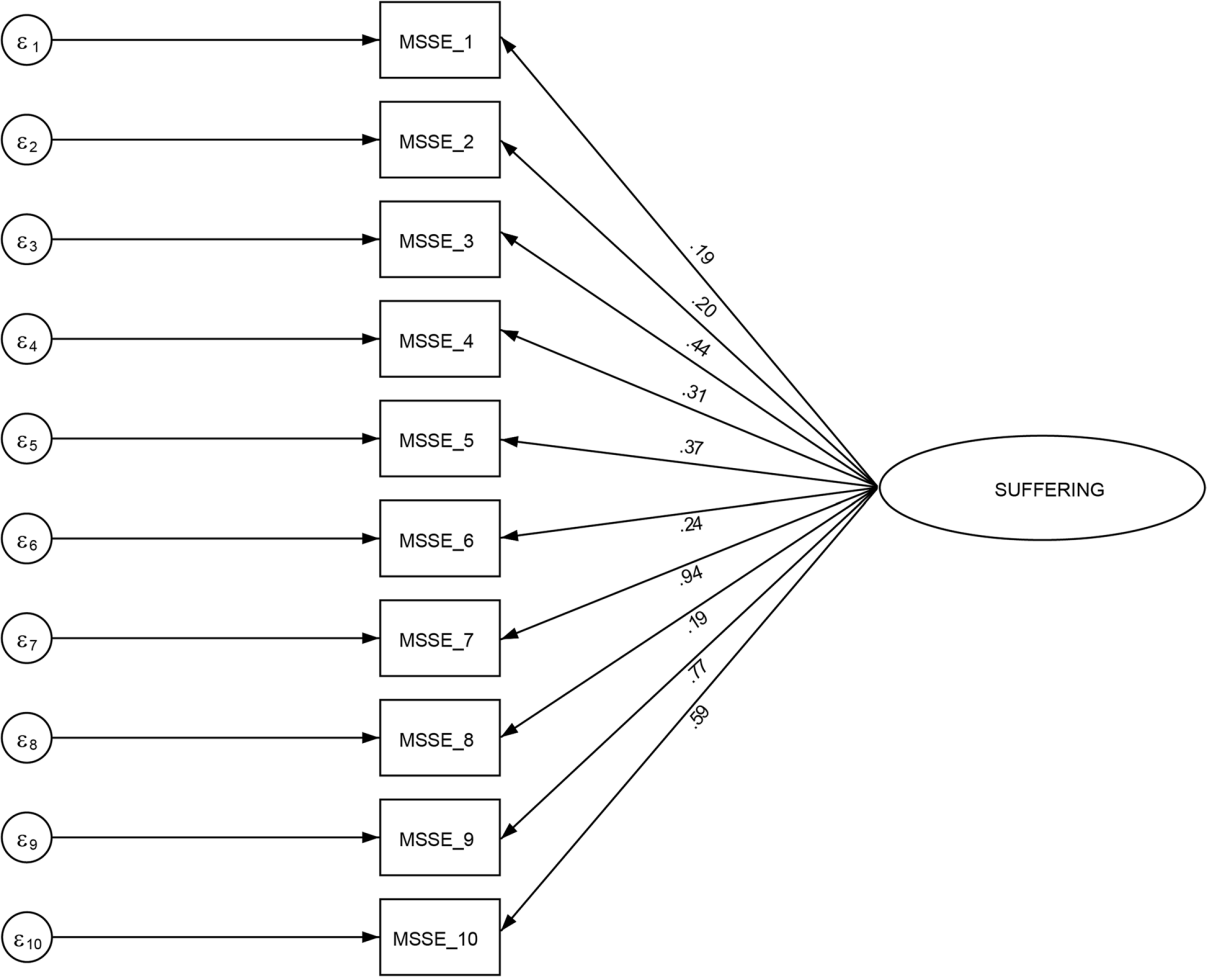
The reflective one-factor model of the German MSSE with error terms e1–e10, and standardized parameter estimates

The CFA showed that the MSSE questionnaire had a low fit to the reflective one-factor structure model, with CFI = 0.71, RMSEA = 0.08, and TLI = 0.64. Figure 3 shows the reflective one-factor model of the German MSSE, with error terms e1–e10 and standardized parameter estimates. Seven of the 10 items underperformed. Only three items had a factor loading of 0.40 or above: pain (0.44), suffering according to medical opinion (0.77), and suffering according to family opinion (0.59).

The level of response coherence to the questionnaire was weak to moderate for seven of the 10 items, as shown by the H values. More precisely, one item was unscalable (“invasive action”), and coherence was weak for four items (“malnutrition,” “eating disorders,” “unstable medical condition,” and “suffering according to family opinion”), moderate for three items (“pain,” “decubitus ulcers,” and “suffering according to medical opinion”), and strong for two items (“not calm,” and “screams”) (see Table 2).

The MSSE scores were moderately correlated with the SM-EOLD scores (*r* = **-**0.44; *p* < 0.05), the physical suffering scores (*r* = 0.41; *p* < 0.05), and the psychological suffering scores (*r* = 0.55; *p* < 0.05).

## Discussion

The present study found that the original English version of the MSSE was based on inadequate theoretical and conceptual underpinnings and an incomplete construction process. Therefore, the MSSE is a poor reflection of the concept of suffering. The instrument could discriminate neither between the different levels of suffering nor between suffering and other distressing symptoms. The German version of the MSSE produced inconsistent and, therefore, incorrect data for PAD living in nursing homes. It had low internal consistency reliability and doubtful validity. Therefore, empirical evidence does not support the intended interpretation and use of the German MSSE scores for needs assessment and decision-making regarding the level of suffering of PAD in the nursing home context. Consequently, we do not recommend its use in this population and setting.

Our theoretical and methodological analysis revealed significant flaws in the scale development process of the original MSSE version, such as a lack of adequate conceptualization and operationalization of the construct of suffering and no assessment of content validity, a vital step in scale development [36]. The theoretical, conceptual, and methodological shortcomings led to confusion about the delimitation of the construct of suffering, including the similarities and differences between suffering and symptom management. These deficiencies weakened the measurement results gained from the original English version and that of the German one, compromising the future applicability of the scale and hindering its generalizability.

In our study, the clustering of PADs at the low level of suffering and the lack of PADs at the high level of suffering suggest that, based on the total scores on the MSSE questionnaire, the PADs could not be sufficiently discriminated from each other concerning their level of suffering. The instrument, therefore, had low discriminatory power.

The values of the KR-20 coefficients indicated low internal consistency reliability. The two subjective items relating to the professional and family estimations of the patient’s suffering had a higher KR-20 coefficient (0.64) than the eight objective items relating to the patient’s health conditions (0.39). These indicator variables appear to have been poorly chosen; thus, there is a need for new variables that are more reflective of the construct of suffering.

The low internal consistency reliability may also have been caused by the response format chosen by the instrument developers. Binary items are easy to answer, but a major shortcoming of binary responses is that each item can have only minimal variability, and any pair of items can only have one of two levels of covariation. With binary items, each item contributes little to the sum of all the elements in the covariance matrix for the individual items due to the limitations in possible variances and covariances. Thus, additional items are needed to obtain the same degree of scale variance if the items are binary [36].

Compared with the coefficient of internal consistency reliability of the total scores of the original version, the scores of the German version were lower, likely reflecting differences in the context and sample composition. Reliability estimates tend to be higher with more heterogeneous populations, which would be expected to have values across the entire range of a measure, as would occur with patients both with and without a condition [49]. In our study, the population was highly homogenous as all PAD had a CPS of 5, except two who had a CPS of 6. The studies conducted using the original English version did not adequately describe the study sample, hindering the interpretability of the scores produced. An in-depth comparison of the context and sample composition between the original English and German versions was impossible (see Table 3).

**Table 3:** Comparison of sample composition and setting of the original English version and the German version of the MSSE questionnaire

Country	Israel (Aminoff et al. 2004) [12]	Israel (Aminoff et al. 2016) [14]	Switzerland
Setting	research and treatment center	geriatric-internal medicine ward of a general tertiary hospital	nursing homes
Sample size	*N* = 103	*N* = 183	*N* = 124
Population	patients with “end-stage dementia”	patients with “end-stage dementia”	patients with advanced dementia
Diagnosis	frequencies not reported	frequencies not reported	64 (51.6%) Alzheimer’s disease, 56 (45.2%) dementia other than Alzheimer’s disease, 4 (3.2%) Alzheimer’s disease and dementia other than Alzheimer’s disease
Mini Mental State Examination (MMSE) [22]	frequencies not reported	frequencies not reported	–
Functional Independence Measure (FIM) [51]	frequencies not reported	frequencies not reported	–
Functional Assessment Staging (FAST) [21]	–	frequencies not reported	–
Cognitive Performance Scale (CPS) [23]	–	–	122 patients (98.4%) had a CPS of 5, 2 (1.6%) had one of 6
Age range	51–96	56–102	55–102
Males	45	73	26
Females	58	110	98
Respondents	two physicians	not reported	95 primary nurses
Time of administration	predeath	predeath	predeath
Recall period	no recall period	no recall period	current situation

As research in cross-cultural gerontology demonstrates [50], different cultural and social contexts affect various phenomena related to aging and influence and shape study objectives and designs. Hence, the different reliability values may have also been caused by different socio-cultural and contextual factors in Israel and Switzerland, leading to variances of concepts and relevance statements about the construct of suffering and how to assess and measure it.

Another reason for the different reliability values may be the different inclusion criteria of the study sample. The inclusion criteria of the ZULIDAD study were a diagnosis of dementia (RAI-MDS items “Alzheimer’s disease” or “dementia other than Alzheimer’s disease”) and a CPS score of 5 or 6, which indicates severe impairment (“advanced dementia”) [23], whereas the instrument developers of the MSSE, as mentioned before in the “background” section, combined the FAS stage 7c [21] and the MMSE score 0/30 [22] (see Table 3).

The CFA showed that seven of the 10 items underperformed as the factor loadings were below 0.30. These items were considered inadequate as they contributed < 10% of the variation of the latent construct being measured. It is recommended that items with factor loadings of 0.40 and above be retained [49, 52]. Only the following three items seemed to be specified correctly: pain, suffering according to medical opinion, and suffering according to family opinion (see Fig. 3). CFA is extremely useful when comparing the original with a translated version of a scale through the use of factor loadings from the original as trial loadings in the second [18]. The instrument developers did not conduct a CFA of the original version and only reported an alpha value [12]. It is insufficient to report only on selected statistical properties of a new instrument and claim that it is reliable and valid. According to Streiner [18], several sources of evidence are required to build the case that the instrument measures what it is supposed and intended to measure. In our study, the MSA was carried out in addition to the CFA to permit further insights into the dimensionality of the MSSE scale. The H values also indicated that the scalability of the MSSE was unsatisfactory.

Compared with the construct validity of the original English version tested with the SM-EOLD scale (*r *= 0.57, *p* < 0.001) [53], the score of the German version was lower. Both global physical suffering and global psychological suffering were moderately correlated with the MSSE. Given the poor measurement properties of the German version of the MSSE, it was impossible to draw valid conclusions from these correlational effects.

Suffering is a complex phenomenon that cannot be easily grasped or measured. Given its many definitions, it is critical to apply specific definitions developed for certain contexts and particular purposes, such as certain types of research or care. Moreover, suffering is multidimensional: it can be influenced by physical and psychological, social, spiritual, and existential factors [6, 48]. As such, a methodologically sound assessment tool should also reflect multidimensional perspectives of suffering, an essential aspect of palliative care [54].

The Suffering Pictogram [55], for instance, is one such robust instrument that could be adopted and tailored to the situation of PAD. It is grounded in elaborate conceptual and theoretical underpinnings. First, the developers carried out a qualitative study among adult palliative care patients to assess their experience with suffering [56]. Based on a thematic network analysis of semi-structured interviews, they deduced the concept of the “suffering threshold,” defined as a unique, subjective, and dynamic point, influenced by various existential and experiential factors, where events and experiences are perceived as suffering. Their existential–experiential model of suffering emphasizes the complex interplay of various factors that inform the formation of suffering. Thus, the authors defined suffering as an unpleasant existential experience that occurs when the individual suffering threshold has been surpassed. Consequently, interventions that modulate the experiential dimension of suffering, in addition to treating unpleasant existential events, may mitigate suffering.

In subsequent research, the instrument developers conducted a validation study of the Suffering Pictogram with 91 palliative care patients [55]. This study found that the Suffering Pictogram was a brief, reliable, and valid instrument to measure experiential suffering in palliative care. A systematic review of instruments assessing suffering in palliative care identified the Suffering Pictogram as the most useful instrument [17]. It was the only measure in which the methodological quality was adequate for content validity, structural validity, internal consistency, and concurrent validity [55].

Although the Suffering Pictogram has only been tested among patients diagnosed with various forms of cancer with palliative care needs, we believe that a modified version of the instrument could be useful to PAD when adopted and tailored to their unique disease profiles, needs, and preferences. Traditionally, the palliative care approach has been associated with people suffering from cancer. Studies have shown that the conceptual foundations of a palliative care approach can be expanded to PAD because they can be considered patients with palliative care needs due to the chronic and life-limiting conditions of the illness [57, 58]. Such a modification of the instrument, which takes the unique condition and situation of PAD into account, requires the involvement of stakeholders, such as patients and their caregivers, researchers, clinicians, instrument developers, advocacy groups, or others, across different stages of the instrument development process. Studies about stakeholder involvement and palliative care have shown that the engagement of stakeholders in research improves the research process and promotes more patient-centered results [59].

Our study did have some limitations. It was based on data collected in 11 nursing homes in Zurich. Therefore, the results are not directly transferable to other settings. Since each PAD was evaluated by a single primary nurse, and given that a single measurement was conducted, we could not report inter-rater, intra-rater, or test–retest reliability. Different levels of education and work experience among the primary nurses may have influenced the administration of the MSSE questionnaire. In our sample, women outnumbered men, an overrepresentation that corresponds with the sex distribution of nursing home residents in Switzerland, three-quarters of whom are women [60].

## Conclusion

We demonstrated that the German version of the MSSE questionnaire did not perform well in the nursing home setting involving PAD. Presumably, due to the insufficient conceptualization and operationalization of the construct of suffering in the original MSSE version, the instrument could discriminate neither between suffering and other distressing symptoms nor between different levels of suffering. Consequently, we do not recommend the use of this instrument in this particular population and setting.

## Data Availability

The data and materials that support the findings of this study are available from the corresponding author, NZ, upon reasonable request.

## Abbreviations

CFA: Confirmatory Factor Analysis
CFI: Comparative Fit Index
CI: Confidence Interval
CPS: Cognitive Performance Scale
FAST: Functional Assessment Staging
FIM: Functional Independence Measure
ISPOR: International Society for Pharmacoeconomics and Outcomes Research
KR-20: Kuder-Richardson Formula 20
M: Mean
MDS: Minimum Data Set
MMSE: Mini Mental State Examination
MSA: Mokken Scale Analysis
MSSE: Mini Suffering State Examination
RAI–MDS: Resident Assessment Instrument–Minimum Data Set
RMSEA: Root Mean Square Error of Approximation
PAD: People with Advanced Dementia
SD: Standard Deviation
SM-EOLD: Symptom Management–End-of-Life with Dementia
TLI: Tucker-Lewis Index
ZULIDAD: Zurich Life and Death with Advanced Dementia
